# Hearing and seeing meaning in noise: Alpha, beta, and gamma oscillations predict gestural enhancement of degraded speech comprehension

**DOI:** 10.1002/hbm.23987

**Published:** 2018-01-30

**Authors:** Linda Drijvers, Asli Özyürek, Ole Jensen

**Affiliations:** ^1^ Radboud University, Centre for Language Studies, Erasmusplein 1 6525 HT Nijmegen The Netherlands; ^2^ Radboud University, Donders Institute for Brain, Cognition, and Behaviour, Montessorilaan 3 6525 HR Nijmegen The Netherlands; ^3^ Max Planck Institute for Psycholinguistics, Wundtlaan 1 6525 XD Nijmegen The Netherlands; ^4^ School of Psychology Centre for Human Brain Health, University of Birmingham, Hills Building Birmingham B15 2TT United Kingdom

**Keywords:** degraded speech, gesture, magnetoencephalography, multimodal integration, oscillations, semantics

## Abstract

During face‐to‐face communication, listeners integrate speech with gestures. The semantic information conveyed by iconic gestures (e.g., a drinking gesture) can aid speech comprehension in adverse listening conditions. In this magnetoencephalography (MEG) study, we investigated the spatiotemporal neural oscillatory activity associated with gestural enhancement of degraded speech comprehension. Participants watched videos of an actress uttering clear or degraded speech, accompanied by a gesture or not and completed a cued‐recall task after watching every video. When gestures semantically disambiguated degraded speech comprehension, an alpha and beta power suppression and a gamma power increase revealed engagement and active processing in the hand‐area of the motor cortex, the extended language network (LIFG/pSTS/STG/MTG), medial temporal lobe, and occipital regions. These observed low‐ and high‐frequency oscillatory modulations in these areas support general unification, integration and lexical access processes during online language comprehension, and simulation of and increased visual attention to manual gestures over time. All individual oscillatory power modulations associated with gestural enhancement of degraded speech comprehension predicted a listener's correct disambiguation of the degraded verb after watching the videos. Our results thus go beyond the previously proposed role of oscillatory dynamics in unimodal degraded speech comprehension and provide first evidence for the role of low‐ and high‐frequency oscillations in predicting the integration of auditory and visual information at a semantic level.

## 1 INTRODUCTION

Successful face‐to‐face communication, especially under adverse listening conditions, needs a weighing and integration of linguistic (e.g., speech) and sensory information (e.g., a co‐speech gesture). To understand how the brain adapts to such audiovisual contexts, a functional network approach is needed in which patterns of ongoing neural activity are considered to allocate computational resources by engaging and disengaging task‐relevant brain areas (Jensen & Mazaheri, 2010; Pfurtscheller & Lopes da Silva, 1999; Siegel, Donner, & Engel, 2012). Suppression of alpha and beta oscillations is often related to the engagement of task‐relevant brain areas, whereas an increase reflects functional inhibition or disengagement (Jensen & Mazaheri, 2010; Klimesch, Sauseng, & Hanslmayr, 2007; Pfurtscheller & Lopes da Silva, 1999). Increases in gamma activity have been proposed to reflect enhanced neuronal computation (Fries, Nikolić, & Singer, 2007; Jensen, Kaiser, & Lachaux, 2007). Previously, oscillatory dynamics in these frequency bands have been studied during auditory comprehension of degraded speech, but it is unknown whether similar mechanisms apply to degraded speech comprehension in the context of meaningful visual input, such as hand gestures. Based on previous research that demonstrated that the magnitude of low‐ and high‐frequency activity can predict the degree of audiovisual integration (Hipp, Engel, & Siegel, 2011), we here investigate whether such oscillatory mechanisms also apply to more realistic settings and audiovisual integration at the semantic level, such as gestural enhancement of degraded speech comprehension.

Listeners routinely process speech and meaningful co‐speech gestures. Behavioral and neuroimaging studies on gesture processing have shown that iconic gestures (e.g., a hand mimicking a drinking action) enhance degraded speech comprehension and are integrated with speech (Beattie & Shovelton, 1999; Drijvers & Ozyürek, 2017; Holle, Obleser, Rueschemeyer, & Gunter, 2010; Obermeier, Dolk, & Gunter, 2012; Özyürek, 2014). fMRI studies have demonstrated that speech–gesture integration involves the LIFG, STS, middle temporal gyrus (MTG), motor, and visual cortex (Dick, Mok, Raja Beharelle, Goldin‐Meadow, & Small, 2014; Green et al., 2009; Straube, Green, Weis, & Kircher, 2012; Willems, Özyürek, & Hagoort, 2007, 2009). However, the spatiotemporal neural dynamics of this integration remain unknown.

Studies on unimodal auditory degraded speech comprehension have demonstrated that parietal alpha power is enhanced when speech is degraded (Becker, Pefkou, Michel, & Hervais‐Adelman, 2013; Drijvers, Mulder, & Ernestus, 2016; Obleser & Weisz, 2012; Weisz, Hartmann, Müller, Lorenz, & Obleser, 2011; Wostmann, Herrmann, Wilsch, & Obleser, 2015). These results were interpreted as reflecting increased auditory cognitive load when the language processing system is inhibited due to degradation. Previous research on gesture processing has reported low‐frequency (2–7 Hz) modulations to emblems (e.g., thumbs‐up gesture occurring without speech) and beat gestures (nonsemantic rhythmic hand flicks) (Biau & Soto‐Faraco, 2015; He et al., 2015), but the spatiotemporal neural dynamics supporting gestural enhancement of speech remain unknown. By using the good temporal and spatial resolution of MEG, we can quantify the spatiotemporal oscillatory dynamics supporting audiovisual integration at a semantic level.

In this study, we presented participants with videos that either contained clear or degraded speech, accompanied by a gesture or not. Our central hypothesis is that gestures enhance degraded speech comprehension and that comprehension relies on an extended network including the motor cortex, visual cortex, and language network to perform this multimodal integration. Here, brain oscillations are assumed to have a mechanistic role in enabling integration of information from different modalities and engaging areas that contribute to this process. We predict that when integration demands increase, we will observe an alpha (8–12 Hz) power suppression in visual cortex, reflecting more visual attention to gestures, and an alpha and beta (15–20 Hz) power decrease in the language network, reflecting the engagement of the language network and a higher semantic unification load (Wang, Zhu, & Bastiaansen, 2012a). Second, we expect an alpha and beta power suppression in the motor cortex, reflecting engagement of the motor system during gestural observation (Caetano, Jousmaki, & Hari, 2007; Kilner, Marchant, & Frith, 2009; Koelewijn, van Schie, Bekkering, Oostenveld, & Jensen, 2008). Last, we predict an increase in gamma power in the language network, reflecting the facilitated integration of speech and gesture into a unified representation (Hannemann, Obleser, & Eulitz, 2007; Schneider, Debener, Oostenveld, & Engel, 2008; Wang et al., 2012b; Willems et al., 2007, 2009).

## 2 MATERIALS AND METHODS

### 2.1 Participants

Thirty‐two Dutch native students of Radboud University (mean age = 23.2, *SD* = 3.46, 14 males) were paid to participate in this experiment. All participants were right‐handed and reported corrected‐to‐normal or normal vision. None of the participants had language, motor or neurological impairment and all reported normal hearing. The data of three participants (two females) was excluded because of technical failure (1), severe eye‐movement artifacts (>60% of trials) (1), and excessive head motion artifacts (>1 cm) (1). The final dataset therefore included the data of 29 participants. All participants gave written consent before they participated in the experiment.

### 2.2 Stimuli

Participants were presented with 160 short video clips of a female actress who uttered a Dutch action verb, which would be accompanied by an iconic gesture or no gesture. These video clips were originally used in a previous behavioral experiment in Drijvers and Ozyürek (2017), where pretests and further details of the stimuli can be found.

The action verbs that were used were all highly frequent Dutch action verbs so that they could easily be coupled to iconic gestures. All videos were recorded with a JVC HY‐HM100 camcorder and had an average length of 2,000 ms (*SD* = 21.3 ms). The actress in the video was wearing neutrally colored clothes and was visible from the knees up, including the face. In the videos where she made an iconic gesture, the preparation of the gesture (i.e., the first video frame that shows movement of the hand) started 120 ms (*SD* = 0 ms) after onset of the video, the stroke (i.e., the meaningful part of the gesture) started on average at 550 ms (*SD* = 74.4 ms), gesture retraction started at 1,380 ms (*SD* = 109.6 ms), and gesture offset at 1,780 ms (*SD* = 150.1 ms). Speech onset started on average at 680 ms (*SD* = 112.54 ms) after video onset, In previous studies this temporal lag was found to be ideal for information from the two channels to be integrated during online comprehension (Habets, Kita, Shao, Ozyurek, & Hagoort, 2011). In 80 of the 160 videos, the actress produced an iconic gesture. All gestures were iconic movements that matched the action verb (see below). In the remaining 80 videos, the actress uttered the action verbs with her arms hanging casually on each side of the body.

It is important to note here that all the iconic gestures were not prescripted by us but were renditions by our actress, who spontaneously executed the gestures while uttering the verbs one by one. As such, these gestures resembled those in natural speech production, as they were meant to be understood in the context of speech, but not as pantomimes which can be fully understood without speech. We investigated the recognizability of all our iconic gestures outside a context of speech by presenting participants with all video clips without any audio, and asked them to name a verb that depicted the video (as part of Drijvers & Ozyürek, 2017). We coded answers as “correct” when a correct answer or a synonym was given in relation to the verb each iconic gesture was produced with by the actor, and as “incorrect” when the verb was unrelated. The videos had a mean recognition rate of 59% (SD ∼ 16%), which indicates that the gestures were potentially ambiguous in the absence of speech, as they are in the case of naturally occurring co‐speech gestures (Krauss, Morrel‐Samuels, & Colasante, 1991). This ensured that our iconic gestures could not be understood fully without speech (e.g., a “mopping” gesture, which could mean either “rowing,” “mopping,” “sweeping,” or “cleaning,” and thus needs the speech to be understood) and that our participants could not disambiguate the degraded speech fully by just simply looking at the gesture and labelling it. Instead, participants needed to integrate speech and gestures for successful comprehension. For further details on the pretesting of our videos, please see Drijvers and Ozyürek (2017).

We extracted the audio from the video files, intensity‐scaled the speech to 70 dB and de‐noised the speech in *Praat* (Boersma & Weenink, 2015). All sound files were then recombined with their corresponding video files. The speech in the videos was presented either clear or degraded (Shannon, Zeng, Kamath, Wygonski, & Ekelid, 1995). As in a previous study on gestural enhancement of degraded speech comprehension (Holle et al., 2010), we determined in our previous behavioral study (Drijvers & Ozyürek, 2017) which degradation level was optimal for gestural information to have the largest impact on enhancing degraded speech comprehension. In going beyond Holle et al., (2010), the only previous study on gestural enhancement of degraded speech, we did not cover the face of the actor and thus studied the gestural enhancement effect in a more natural context. This allowed us to investigate how gestures enhance degraded speech comprehension on top of the context of the (phonological) cues that are conveyed by visible speech. In Drijvers and Ozyürek (2017), participants completed a free‐recall task where they were asked to write down the verb they heard in videos that were either presented in 2‐band noise‐vocoding, 6‐band noise‐vocoding, clear speech, and visual‐only conditions that did not contain any audio.

Our previous results from Drijvers and Ozyürek (2017) demonstrated that listeners benefitted from gestural enhancement most at a 6‐band noise‐vocoding level. At this noise‐vocoding level, auditory cues were still reliable enough to benefit from both visual semantic information and phonological information from visible speech. However, in 2‐band noise‐vocoding, listeners could not benefit from the phonological information that was conveyed by visible speech to couple the visual semantic information that was conveyed by the gesture. Instead, in 2‐band noise‐vocoding, the amount of correct answers was as high in the visual only condition that did not have audio.

In addition to clear speech, we thus created a 6‐band noise‐vocoding version of each clear audio file that was then recombined with the video, using a custom‐made script in *Praat*, by bandpass filtering each sound file between 50 and 8,000 Hz and dividing the speech signal by logarithmically spacing the frequency bands between 50 and 8,000 Hz. In more detail, this resulted in cutoff frequencies of 50, 116.5, 271.4, 632.5, 1,473.6, 3,433.5, and 8,000 Hz. We used half‐wave rectification to extract the amplitude envelope of each band and multiplied the amplitude envelope with the noise bands before recombining the bands to form the degraded speech signal. The sound of the videos was presented through MEG‐compatible air tubes.

**Figure 1:**
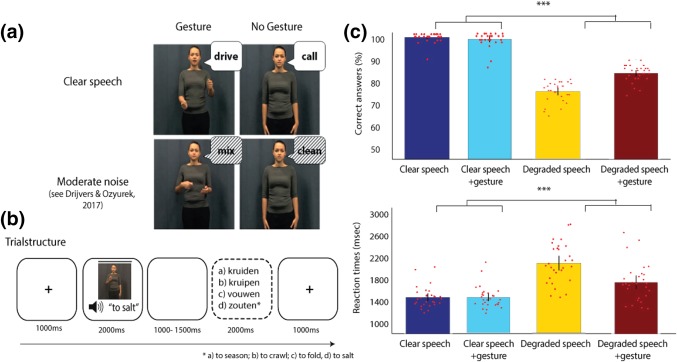
(a) Illustration of the different conditions. (b) Trial structure. (c) Upper panel: percentage of correct answers per condition. Error bars represent *SD*. ***p < .01. Lower panel: reaction times (in milliseconds) per condition. Error bars represent *SD*. ***p < .01. Red dots represent individual participant's data [Color figure can be viewed at http://wileyonlinelibrary.com]

In total, we included four conditions in our experiment: a clear speech only condition (C), a degraded speech only condition (D), a clear speech + iconic gesture condition (CG), and a degraded speech + iconic gesture condition (DG) (Figure 1a). All four conditions contained 40 videos, and none of the verbs in the videos overlapped. Note that we did not follow the design described in Drijvers and Ozyürek (2017), as using eleven conditions would have resulted in a very low number of trials per condition for source analyses.

Finally, to assess the participants’ comprehension of the verbs, we presented participants with a cued‐recall task (see for details below) instead of the free‐recall task that was used in Drijvers and Ozyürek (2017), as a free‐recall task would have caused too many (motion) artifacts for the MEG analyses.

### 2.3 Procedure

Participants were tested in a dimly‐lit magnetically shielded room and seated 70 cm from the projection screen. All videos were projected onto a semi‐translucent screen by back‐projection using an EIKI LC‐XL100L projector with a resolution of 1,650 × 1,080 pixels. The stimuli were presented full screen using Presentation software (Neurobehavioral Systems, Inc.) In the experiment, participants were asked to attentively listen and watch the videos. Each trial started with a fixation cross (1,000 ms), followed by the video (2,000 ms), a short delay (1,000–1,500 ms, jittered), followed by a cued‐recall task, After watching the videos, participants were asked to identify what verb they had heard in the last video. Participants could indicate their choice by a right‐hand button press on a 4‐button box, where the 4 buttons represented the answering options for either a, b, c, or d. These answering options always contained a phonological distractor, a semantic distractor, an unrelated answer, and the correct answer. For example, the correct answer could be “kruiden” (to season), the phonological distractor could be “kruipen” (to crawl), the semantic distractor, which would fit with the gesture, could be “zouten” (to salt), and the unrelated answer could be “vouwen” (to fold) (Figure 1). The cued‐recall task ensured that participants were paying attention to the videos, and to check whether participants behaviorally resolved the verbs. Furthermore, the semantic competitors were included to investigate whether participants were focusing on the gesture only in the degraded speech conditions. We predicted that if this was indeed the case, they would choose the semantic competitors if they solely zoomed in on the gesture and ignored the degraded speech. After participants indicated their answers, a new trial would start after 1,500 ms (Figure 1b). Participants were asked not to blink during the videos, but to blink after they had answered the question in the cued‐recall task.

Brain activity was recorded with MEG during the whole task, which consisted of 4 blocks of 40 trials. Participants had a self‐paced break after each block. The whole experiment lasted about one hour, including preparation of the participant and instruction of the task. All participants were presented with a different pseudo‐randomization of the stimuli, with the constraint that a specific condition (e.g., two trials of DG) could not be presented more than twice in a row.

### 2.4 Experimental design and statistical analyses: MEG data acquisition

MEG was recorded by using a 275‐channel axial gradiometer CTF MEG system. An online low‐pass filter with a cutoff at 300 Hz was applied, and the data were digitized at 1.2 kHz and stored for offline analyses. Additionally, we recorded participants' eye gaze by using an SR Research Eyelink 1000 eye tracker, to monitor fixation during the task. Participants' electrocardiogram (ECG) and horizontal and vertical electrooculogram (EOG) were recorded for artifact rejection purposes. To measure and monitor the participants' head position with respect to the gradiometers, we placed three coils at the nasion and left/right ear canal. We monitored head position in real time (Stolk, Todorovic, Schoffelen, & Oostenveld, 2013). After the experimental session, we recorded structural magnetic resonance images (MRI) from 22 out of 32 subjects using a 1.5 T Siemens Magnetom Avanto system with markers attached in the same position as the head coils, to align the MRIs with the MEG coordinate system in our analyses.

### 2.5 MEG data analyses: Preprocessing and time–frequency representations of power

We analyzed the MEG data using FieldTrip (Oostenveld, Fries, Maris, & Schoffelen, 2011), an open‐source MATLAB toolbox. First, the MEG data were segmented into trials starting 1 s before and ending 3 s after the onset of the video. The data were demeaned and a linear trend was fitted and removed. Line noise was attenuated using a discrete Fourier transform approach at 50 and 100 Hz (first harmonic) and 150 Hz (second harmonic). We applied a third‐order synthetic gradiometer correction (Vrba & Robinson, 2001) to reduce environmental noise, and rejected trials (on average 6.25%) that were contaminated by SQUID jump artifacts or muscle artifacts using a semi‐automatic routine. Subsequently, we applied independent component analysis (Bell & Sejnowski, 1995; Jung et al., 2000) to remove eye movements and cardiac‐related activity. Finally, the data were inspected visually to remove artifacts that were not identified by these rejection procedures and resampled the data to 300 Hz to speed up the subsequent analyses (average number of trials per participant discarded: 9.97, *SD* = 3.08). To facilitate interpretation of the MEG data, we calculated synthetic planar gradients, as planar gradient maxima are known to be located above neural sources that may underlie them (Bastiaansen & Knösche, 2000). Here, the axial gradiometer data were converted to orthogonal planar gradiometer pairs, after which power was computed, and then the power of the pairs was summed.

The calculation of time–frequency representations (TFRs) of power per condition was carried out in two frequency ranges to optimize time and frequency resolution. First, we calculated the TFRs of the single trials between 2 and 30 Hz, by applying a 500 ms. Hanning window in frequency steps of 1 Hz and 50 ms time steps. In the 30–100 Hz frequency range, a multitaper (discrete prolate spheroidal sequences) approach was used (Mitra & Pesaran, 1999), by applying a 500 ms window length, 2 Hz frequency steps, 50 ms time steps, and 5 Hz frequency smoothing. To capture the gestural enhancement effect, we compared the differences in *Degraded Speech + Gesture* and *Degraded Speech* to the difference in *Clear Speech + Gesture* and *Clear Speech*. The four conditions (C, D, CG, DG) were averaged separately for each participant. TFRs were then log10 transformed and the difference between the conditions (D vs C, DG vs CG, DG vs D, and CG vs C) was calculated by subtracting the log10 transformed power (“log ratio,” e.g., log10(DG) − log10(D)). To calculate the effect of gestural enhancement, we compared the differences between DG versus D and CG versus C (i.e., (log10(DG) − log10(D)) − (log10(CG) − log10(C)). Our time window of interest was between 0.7 and 2.0, which corresponded to the speech onset of the target word until the offset of the video. The range of our frequency bands of interest were selected on the basis of our hypotheses and a grand average TFR of all conditions combined.

### 2.6 MEG data analysis: Source analyses

Source analysis was performed using dynamic imaging of coherent sources (DICS; Gross et al., 2001) as a beamforming approach. We based our source analysis on the data recorded from the axial gradiometers. DICS computes a spatial filter from the cross‐spectral density matrix (CSD) and a lead field matrix. We obtained individual lead fields for every participant by spatially co‐registering the individual anatomical MRI to sensor space MEG data by identifying the anatomical markers at the nasion and the two ear canals. We then constructed a realistically shaped single‐shell head model on the basis of the segmented MRI for each participant, divided the resulting brain volume into a 10 mm spaced grid and warped it to a template brain (MNI). We also used the MNI template brain for the participants who did not come back for the MRI scan.

The CSD was calculated on the basis of the results of the sensor‐level analyses: For the alpha band, we computed the CSD between 0.7–1.1, 1.1–1.5, and 1.6–2.0 s at 10 Hz with ±2.5 Hz frequency smoothing. For the beta band, we computed the CSD between 1.3 and 2.0 s, centered at 18 Hz with ±4 Hz frequency smoothing and for the gamma band between 1.0 and 1.6 s, between 65 and 80 Hz, with 10 Hz frequency smoothing. A common spatial filter containing all conditions was calculated and the data were projected through this filter, separately for each condition. The power at each gridpoint was calculated by applying this common filter to the conditions separately, and was then averaged over trials and log10 transformed. The difference between the conditions was again calculated by subtracting the log‐power for the single contrasts, and interaction effects were obtained by subtracting the log‐power for the two contrasts. Finally, for visualization purposes, the grand average grid of all participants was interpolated onto the template MNI brain.

### 2.7 Cluster‐based permutation statistics

We performed cluster‐based permutation tests (Maris & Oostenveld, 2007) to assess the differences in power in the sensor and source‐level data. The statistical tests on source‐level data were performed to create statistical threshold masks to localize the effects we observed on sensor level. A nonparametric permutation test together with a clustering method was used to control for multiple comparisons. First, we computed the mean difference between two conditions for each data sample in our dataset (sensor: each sample for sensor TFR analysis, source: *x*/*y*/*z* sample for source space analysis). Based on the distribution that is obtained after collecting all the difference values for all the data samples, the observed values were thresholded with the 95th percentile of the distribution, which were the cluster candidates (i.e., mean difference instead of *t* values), and randomly reassigned the conditions in participants 5,000 times to form the permutation distribution. For each of these permutations, the cluster candidate who had the highest sum of the difference values was added to the permutation distribution. The actual observed cluster‐level summed values were compared against the permutation distribution, and those clusters that fell in the highest or lowest 2.5% were considered significant. For the interaction effects, we followed a similar procedure and compared two differences to each other. Note that we do not report effect sizes for these clusters as there is not a simple way of translating the output of the permutation testing to a measure of effect size.

### 2.8 The relation between alpha, beta, and gamma oscillations and behavioral cued‐recall scores

We further tested whether power modulations in the alpha, beta, and gamma frequency band were related to the participants' individual scores on the cued‐recall task. Specifically, we quantified the individual's power modulation in each frequency band by averaging the power modulation over time points, frequencies, and sensors in significant clusters of the interaction effects, resulting in an individual's modulation score per frequency band. Similarly, we calculated an interaction score for gestural enhancement on the behavioral task by comparing the difference in the percentage of correct answers of DG‐D to the difference in CG‐C, resulting in the amount of behavioral enhancement per participant. We then obtained Spearman correlation between this score and the power modulation per frequency band. As our hypotheses stated that the gestural enhancement effect would be supported by an alpha/beta suppression and a gamma power increase, we used one‐tailed *t* tests to test for this correlation.

## 3 RESULTS

Participants were presented with videos that contained a gesture or no gesture, and listened to action verbs that were degraded or not (Figure 1a,b). After each presentation, participants were prompted by a cued‐recall task and instructed to identify which verb they had heard in the videos (Figure 1b). We defined the “gestural enhancement” as the interaction between the occurrence of a gesture (present/not present) and speech quality (clear/degraded), and predicted that the enhancement would be largest when speech was degraded and a gesture was present. Brain activity was measured using whole‐head MEG throughout the whole experiment. The time interval of interest for the analysis was always 0.7–2.0s, from speech onset until video offset (Figure 3a).

### 3.1 Gestural enhancement is largest during degraded speech comprehension

Our behavioral data revealed, in line with previous behavioral studies (Drijvers & Ozyürek, 2017; Holle et al., 2010), that gesture enhanced speech comprehension most when speech was degraded. The percentage of correct answers in the cued‐recall task were analyzed by applying a repeated measures ANOVA with the factors Noise (clear speech vs degraded speech) and Gesture (present vs not present). This revealed a main effect of Noise, indicating that when speech was clear, participants were better able to identify the verb than when the speech was degraded (*F*(1,28) = 83.79, *p* < .001, η^2^ = .75). A main effect of Gesture (*F*(1,28) = 7.93, *p* = .009, η^2^ = .22), demonstrated that participants provided more correct answers when a gesture was present. Our main finding was a significant interaction between Noise and Gesture (*F*(1,28) = 17.12, *p* < .001, η^2^ = .38), which indicated that gestures facilitated speech comprehension in particular in the degraded condition. A repeated measures ANOVA applied to the reaction times with the factors Noise (clear speech vs. degraded speech) and Gesture (present vs. not present) revealed a main effect of Noise, indicating that when the speech signal was clear, participants responded faster (*F*(1,28) = 93.02, *p* < .001, η^2^ = .77). A main effect of Gesture (*F*(1,28) = 5.66, *p* = .024, η^2^ = .17; Figure 1c), indicated that when a gesture was present, participants responded faster. The data revealed an interaction between Noise and Gesture (*F*(1,28) = 12.08, *p* < .01, η^2^ = .30), which indicated that when speech was degraded and a gesture was present, participants were quicker to respond.

It should be acknowledged that these results seem attenuated as compared to the results from Drijvers and Ozyürek (2017). In this experiment, we for example reported a behavioral benefit when comparing DG to D of ∼40%, as compared to approximately 10% in the current study. This can be explained by the type of task we used. In the free‐recall task, participants were unrestricted in their answers, whereas in the cued‐recall task, recognition was easier. This especially had an influence on the increased recognition of the verbs in the D condition, where participants were more able to correctly identify the verb when the answers were cued. Nevertheless, we see a similar pattern (DG‐D) in the data of this study and Drijvers and Ozyürek (2017). Note that the low amount of errors in the current study, and the low amount of semantic errors (∼3%, *SD* = 1.6%), confirmed that the participants did not solely attend to the gesture for comprehension in the DG condition.

### 3.2 Alpha power is suppressed when gestures enhance degraded speech comprehension

**Figure 2:**
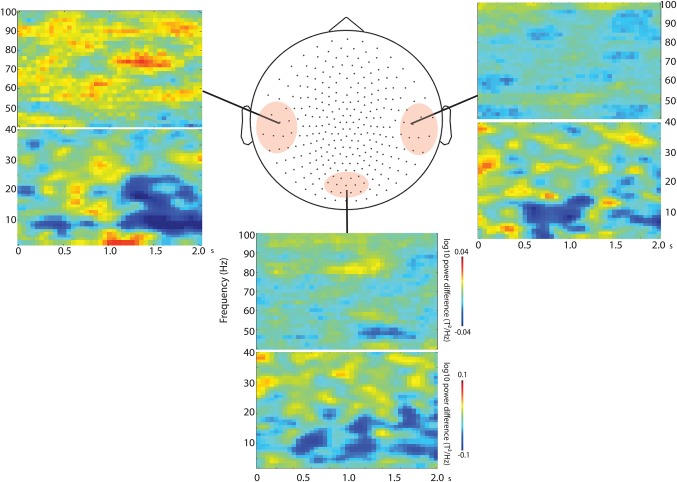
Time–frequency representations (TFRs) of power of the interaction effect between noise and gesture (Gestural enhancement effect) over three selected groups of representative sensors [Color figure can be viewed at http://wileyonlinelibrary.com]

Next we asked how oscillatory dynamics in the alpha band were associated with gestural enhancement of degraded speech comprehension. To this end, we calculated the time–frequency representations (TFRs) of power for the individual trials. These TFRs of power were then averaged per condition. The interaction was calculated as the log‐transformed differences between the conditions. Figure 2 presents the TFRs of power in response to gestural enhancement at representative sensors over the left temporal, right temporal, and occipital lobe. We observed a suppression of alpha power in the right temporal lobe at speech onset when speech was degraded and a gesture was presented, suggesting engagement of right‐temporal areas in an early time window. Additionally, we predicted that alpha would be suppressed over visual regions to allow for more visual attention to the gestures when speech was degraded. In line with our hypotheses, the TFR over occipital regions clearly showed a suppression of alpha power (8–12 Hz) over the full time interval. Last, the TFR of the left temporal lobe revealed a strong alpha suppression from 1.1 s until the end of the video, suggesting engagement of the language system.

**Figure 3:**
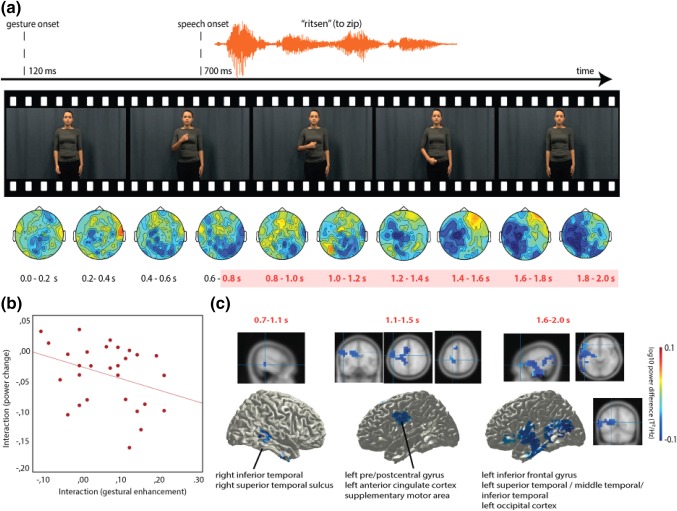
(a) Illustration of the structure of the videos. Lower panel: Topographical distribution of oscillatory alpha power of the gestural enhancement effect in 200 ms time bins from the start of the video until the end of the video. Shaded time windows denote significant clusters in sensor‐level analyses. (b) Individual's alpha power modulations as a function of individual's gestural enhancement scores on the cued‐recall task. (c) Source‐localized results of the interaction effect in the alpha‐band, masked by statistically significant clusters [Color figure can be viewed at http://wileyonlinelibrary.com]

To get more insight into these effects in space and time, we visualized the topographical distribution of the interaction in the alpha band over time (Figure 3a). The top panel represents structure of the videos, and the lower panel shows the topographical distributions over time of alpha power. These topographies reveal an early suppression of alpha power in the right temporal lobe (0.7–1.1 s), followed by an alpha suppression over left central regions (1.1–1.5 s) and left‐temporal and occipital regions (1.6–2.0 s).

Sensor‐level analyses of the interaction effect confirmed a larger suppression of alpha power in in response to DG‐D as compared to CG‐C, indicating that when speech is degraded and a gesture is present, alpha power was more suppressed. A cluster randomization approach controlling for multiple comparisons in time and space revealed one negative cluster (0.7–2.0 s: *p* < .001, summed cluster statistic = −53.3).

Finally, we correlated the individual alpha power modulation with individual behavioral scores on the cued‐recall task, which revealed that the more a listener's alpha power was suppressed, the more a listener showed an effect of gestural enhancement during degraded speech comprehension (Spearman's rho = −.465, *p* = .015, one‐tailed, FDR corrected Figure 2b).

### 3.3 Alpha suppression reveals engagement of rSTS, LIFG, language network, motor, and visual cortex

To determine the underlying sources of this alpha power modulation during gestural enhancement of degraded speech comprehension, we used a frequency‐domain spatial beamformer technique (DICS; Gross et al., 2001). Instead of calculating the source of the negative cluster that was found in the sensor analysis over the whole time window (0.7–2.0 s), we divided this time window over three separate time windows, due to the distinct spatial sources that differed over time (0.7–1.1, 1.1–1.5, and 1.6–2.0 s; see topographical plots in Figure 2). Furthermore, we applied a cluster‐randomization approach to the source data to find a threshold for when to consider the source estimates reliable (note that the cluster‐approach at sensor level constitutes the statistical assessment; not the source level approach). Figure 3c shows that in the 0.7–1.1 s window, the source of the alpha power interaction was localized to the rSTS and to a lesser extent, the right inferior temporal lobe. This suggests engagement of the rSTS during gestural enhancement of degraded speech comprehension immediately after speech onset (one negative cluster, *p* = .042, summed cluster statistic = −9.64). In the 1.1–1.5 s time window, the source of the alpha effect was localized to the left pre‐ and postcentral gyrus, and the supplementary motor area (SMA) and (anterior) cingulate cortex (ACC) (one negative cluster, *p* = .016, summed cluster statistic = −18.58). The axial plots in the second time window in Figure 3c reveal that this alpha effect extends over a large part of the motor cortex and cingulate cortex. The alpha effect in the 1.6–2.0 s time window (one negative cluster, *p* = .002; summed cluster statistic = −26.65) was estimated in the LIFG, STG, MTG, ITG, and left occipital cortex. These results suggest engagement of an initially right lateralized source, followed by left central, temporal, and occipital sources during gestural enhancement of degraded speech comprehension. For comparisons of the single contrasts, please see Supporting Information, S1.

### 3.4 Beta power is suppressed when gestures enhance degraded speech comprehension

**Figure 4:**
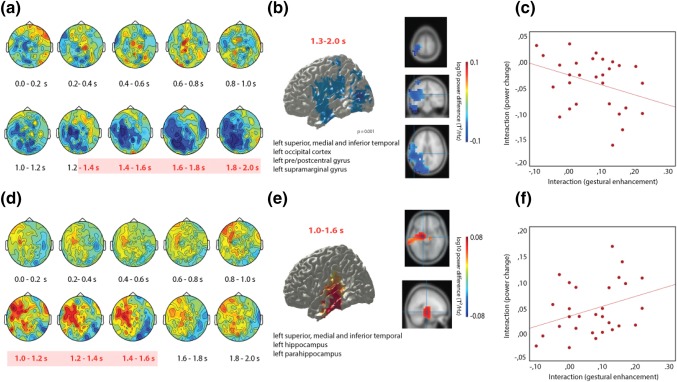
(a) Topographical distribution of oscillatory beta power of the gestural enhancement effect in 200 ms time bins from the start of the video until the end of the video. Shaded time windows denote significant clusters in sensor‐level analyses. (b) Source‐localized results of the interaction effect in the beta‐band, masked by statistically significant clusters. (c) Individual's beta power modulations as a function of individual's gestural enhancement scores on the cued‐recall task. (d) Topographical distribution of oscillatory gamma power of the gestural enhancement effect in 200 ms time bins from the start of the video until the end of the video. Shaded time windows denote significant clusters in sensor‐level analyses. (e) Source‐localized results of the interaction effect in the gamma‐band, masked by statistically significant clusters. (f) Individual's gamma power modulations as a function of individual's gestural enhancement scores on the cued‐recall task [Color figure can be viewed at http://wileyonlinelibrary.com]

Next, we investigated whether gestural enhancement induced modulations of oscillatory beta power. The TFRs of the interaction effect in Figure 2 revealed a left‐lateralized beta power suppression [15–20 Hz] from 1.3 to 2.0s, possibly extending to more posterior areas. We first plotted the topographical distribution of beta power over time to further investigate the spatiotemporal course of this effect (Figure 4a) and observed a larger beta power suppression from ∼1,000 ms, when the meaningful part of the gesture commences, which extended until the end of the video. Sensor‐level analyses of the interaction effect confirmed a stronger suppression of beta power in DG‐D than in CG‐C from 1.3 to 2.0s (negative cluster *p* < .001, summed cluster statistic = −32.85). We correlated the beta power modulation per participant with individual scores on the cued‐recall task, which demonstrated a significant relationship between the amount of beta power suppression and the benefit an individual had from gestures when speech was degraded (i.e., gestural enhancement, see Figure 4c) (Spearman's rho −.352, *p* = .03, one‐tailed, FDR corrected).

### 3.5 Beta power suppression reflects engagement of LIFG, left motor, SMA, ACC, left visual, and left temporal regions

We then localized the gestural enhancement effect to test our hypotheses on the sources for this effect (Figure 4b). This analysis demonstrated that the stronger suppression of beta power was localized (one negative cluster, 1.3–2.0 s *p* < .001; summed cluster statistic = −26.13) in the left pre‐ and postcentral gyrus, ACC, SMA, LIFG, but was also extended to more temporal sources, such as the left superior, medial and inferior temporal regions, the left supramarginal gyrus, and the visual cortex. Note that the observed sources partially overlap with the sources in the alpha band (Figure 3c). This might suggest that some of the beta sources are explained by higher harmonics in the alpha band. Note however that there is a clearer motor beta effect in the beta band than the alpha band. The cluster in the beta band is extending over a part of the motor cortex that corresponds to the hand region of the primary motor cortex, whereas the alpha effect in Figure 3b is more pronounced over the arm–wrist region. This suggests that this beta power effect is possibly more motor‐related than the observed alpha effect. For comparisons of the single contrasts, please see Supporting Information, S2.

### 3.6 Gamma power is enhanced when gestures aid degraded speech comprehension

Finally, we investigated whether gestural enhancement induced reliable modulations of oscillatory power in the gamma band. The TFRs in Figure 2 revealed a left‐temporal increase in gamma band power at 65–80 Hz. We plotted the topographical distributions of this interaction in the gamma band to investigate the spatiotemporal profile (Figure 4d). These topographical plots showed a similar gamma power increase in the 1.0–1.6 s interval. Cluster‐based permutation tests on sensor‐level data of the gestural enhancement effect revealed that this effect was larger in DG‐D than in CG‐C (one positive cluster, *p* = .016; summed cluster statistic = 9.56). Interestingly, these effects occur exactly when the meaningful part of the speech and the meaningful part of the gesture are unfolding. A listener's individual gamma power increase correlated positively with how much this listener could benefit from the semantic information conveyed by a gesture to enhance degraded speech comprehension (Figure 4f, Spearman's rho = .352, *p* = .03, one‐tailed, FDR corrected).

### 3.7 Gamma power increases in left‐temporal and medial temporal areas suggest enhanced neuronal computation during gestural enhancement of degraded speech comprehension

We hypothesized that gamma power would be increased over LIFG and pSTS/STG/MTG, suggesting a facilitated integration of the visual and auditory information into a unified representation (Hannemann et al., 2007; Schneider et al., 2008; Wang et al., 2012b). We therefore conducted source‐level analyses to use as a statistical threshold for estimating the source of the observed sensor‐level effect. In line with our hypotheses, this increase in gamma band power was observed over left superior, medial and inferior temporal regions (Figure 4e, one positive cluster, *p* = .01, summed cluster statistic = 20.76), suggesting neuronal computation when speech is degraded and a gesture is present. This gamma power increase was also identified in sources in deeper brain structures, such as the medial temporal lobe which will be further discussed in Section 4.5. For comparisons of the single contrasts, please see Supporting Information, S3.

## 4 DISCUSSION

This study investigated oscillatory activity supporting gestural enhancement of comprehension of degraded speech, to gain insight into the spatiotemporal neuronal dynamics associated with semantic audiovisual integration. When gestures enhanced degraded speech comprehension, we observed a stronger alpha and beta power suppression, suggesting engagement of the hand‐area of the motor cortex, the extended language network (LIFG/pSTS/STG/MTG), medial temporal lobe, and occipital regions. In the alpha band, this effect displayed a spatiotemporal shift from rSTS, to left motor cortex, ACC, the language network, and visual cortex. The stronger suppression in the beta band occurred in the left hand area of the primary motor cortex, SMA, ACC, LIFG, left‐temporal, and visual cortex. Gestural enhancement was associated with enhanced gamma power over left‐temporal and medial‐temporal lobe regions. All individual oscillatory power modulations significantly correlated with an individual's behavioral score, demonstrating that individual oscillatory power modulations predict how much a listener could benefit from the semantic information conveyed by gestures to enhance degraded speech comprehension. Below we interpret these findings and discuss the putative role of the oscillatory dynamics in task‐relevant brain areas during gestural enhancement of degraded speech.

### 4.1 Early alpha suppression reflects engagement of rSTS to optimally process the upcoming word

In an early time window (0.7–1.1 s), we observed stronger alpha suppression in the rSTS when gestures enhanced degraded speech. In fMRI studies on auditory degraded speech perception, the rSTS has shown to be sensitive to spectral fine‐tuning (Scott, 2000; Zatorre, Belin, & Penhune, 2002) and pitch contours (Gandour et al., 2004; Kotz et al., 2003). In the (audio)visual domain, fMRI and EEG studies have demonstrated that the rSTS responds to motion and intentional action, and bilateral STS showed increased activation during audiovisual integration under adverse listening conditions (Saxe, Xiao, Kovacs, Perrett, & Kanwisher, 2004; Schepers, Schneider, Hipp, Engel, & Senkowski, 2013). The rSTS is possibly engaged because the semantic information conveyed by the gesture is most informative during degraded speech, causing listeners to focus more on the preparation of a gesture early in the video. The larger engagement of the rSTS might thus reflect increased comprehension of the gesture when speech is degraded.

### 4.2 Listeners engage their motor system most when a gesture is presented in degraded speech

During gestural enhancement of degraded speech comprehension, an alpha (1.1–1.5 s) and beta (1.3–2.0 s) power suppression were observed over the hand motor area, primary motor cortex, and SMA. This suggests that the involvement of the motor system might be modulated by the listener's interpretation of ongoing speech perception, resulting in the largest engagement when speech is degraded. This suggests that engaging the motor system during gestural observation in degraded speech might be a result of aiding interpretation, rather than simple mirroring of the observed action, or mere involvement limited to the production and perception of linguistic or sensory information (see for debate, e.g., Toni, de Lange, Noordzij, & Hagoort, 2008). Rather, our results suggest that listeners might simulate the gesture more when speech is degraded, possibly to extract the meaning of the gesture to aid in interpreting the degraded speech, which is in line with previous studies on action observation (van Elk, van Schie, Zwaan, & Bekkering, 2010; Klepp, Niccolai, Buccino, Schnitzler, & Biermann‐Ruben, 2015; Weiss & Mueller, 2012) and embodied cognition (Pulvermuller, Hauk, Nikulin, & Ilmoniemi, 2005; Barsalou, 2008).

### 4.3 The ACC engages in implementing strategic processes to use gestural information to understand degraded speech

The sources of the alpha and beta power suppression described in Section 4.2, both extended to the ACC. Caution should be taken when interpreting deep sources like the ACC when using MEG; however, our results are consistent with related brain imaging findings. Previous research using fMRI reported enhanced activity in the ACC when modality‐independent tasks increased in difficulty, when listeners attended to speech, and during degraded speech comprehension (Eckert et al., 2009; Erb, Henry, Eisner, & Obleser, 2013; Peelle, 2017), suggesting that these areas are involved in attention‐based performance monitoring, executive processes and optimizing speech comprehension performance (Vaden et al., 2013). Additionally, previous research has reported that the ACC might subserve an evaluative function, reflecting the need to implement strategic processes (Carter et al., 2000). As the current effect occurs when the meaningful part of the speech and gesture are unfolding, we interpret the alpha and beta power suppression as engagement of the ACC to enhance attentional mechanisms and possibly strategically shift attention to gestures, and allocate resources to increase the focus on semantic information conveyed by the gesture.

### 4.4 A left‐lateralized network including IFG, pSTS/MTG, ITG, and STG is most engaged when gestures enhance degraded speech comprehension

During gestural enhancement of degraded speech, an alpha (1.6–2.0 s) and beta (1.3–2.0 s) power suppression were observed in LIFG and left posterior temporal regions (pSTS/MTG, ITG, STG). Activation of left posterior temporal regions has been proposed to be involved in retrieving lexical‐semantic, phonological, morphological, and syntactical information (Hagoort, 2013; Lau, Phillips, & Poeppel, 2008). The LIFG is thought to be involved in unification operations from building blocks that are retrieved from memory and selection of lexical representations and the unification of information from different modalities (Hagoort, 2013). A beta power suppression in LIFG has been related to a higher unification load that requires a stronger engagement of the task‐relevant brain network (Wang et al., 2012a). In line with this, we suggest that the larger alpha and beta power suppression in LIFG reflects engagement during the unification of gestures with degraded speech. We tentatively propose that this larger engagement might facilitate lexical retrieval processes by unifying speech and gesture. Here, the semantic information of the gesture might facilitate lexical activation of the degraded word, which simultaneously engages the language network in this process.

Note that this tentative explanation is also supported by analyses conducted over the single contrasts: In line with previous auditory literature (Obleser et al., 2012; Weisz et al., 2011) we observed enhanced alpha power in response to degraded speech, which has been suggested, in line with the functional inhibition framework, to possibly act as a “gating mechanism” toward lexical integration, reflecting neural oscillators that keep alpha power enhanced to suppress erroneous language activations. However, we observed a larger alpha *suppression* in conditions that contained gestural information. We argue that the occurrence of a gesture thus seems to reverse the inhibitory effect that degraded speech imposes on language processing, by engaging task‐relevant brain regions when the semantic information of the gesture facilitates lexical activation, and thus requires less suppression of potentially erroneous activations in the mental lexicon.

### 4.5 Semantic information from gestures facilitates a matching of degraded speech with top–down lexical memory traces in the MTL

Gamma power was most enhanced when the meaningful part of the gesture and degraded speech were unfolding. This enhancement was estimated in the left (medial) temporal lobe. Enhanced gamma activity has been associated with the integration of object features, the matching of object specific information with stored memory contents and neuronal computation (Herrmann, Munk, & Engel, 2004; Tallon‐Baudry & Bertrand, 1999). In line with this, the observed gamma effect in the left temporal lobe might reflect cross‐modal semantic matching processes in multisensory convergence sites (Schneider et al., 2008), where active processing of the incoming information facilitates an integration of the degraded speech signal and gesture. Next to left‐temporal sources, enhanced gamma power was localized in deep brain structures, such as the medial temporal lobe. We tentatively propose that the observed gamma increases in medial temporal regions reflect that the semantic information conveyed by gestures can facilitate a matching process with lexical memory traces that aids retrieval of the degraded input.

### 4.6 Engagement of the visual system reflects that listeners allocate visual attention to gestures when speech is degraded

We observed the largest alpha (1.6–2.0 s) and beta (1.3–2.0 s) suppression during gestural enhancement of degraded speech. We interpret these larger suppressions as engagement of the visual system and allocation of resources to visual input (i.e., gestures), especially when speech is degraded.

### 4.7 Individual oscillatory power modulations correlate with a listener's individual benefit of a gesture during degraded speech comprehension

We demonstrated a clear relationship between gestural enhancement effects on a behavioral and neural level: The more an individual listener's alpha and beta power were suppressed and the more gamma power was increased, the more a listener benefitted from the semantic information conveyed by a gesture during degraded speech comprehension. This gestural benefit was thus reflected in neural oscillatory activity and demonstrates the behavioral relevance of neural oscillatory processes.

## 5 CONCLUSIONS

The present work is the first to elucidate the spatiotemporal oscillatory neural dynamics of audiovisual integration in a semantic context and directly relating these modulations to an individual's behavioral responses. When gestures enhanced degraded speech comprehension, alpha and beta power suppression suggested engagement of the rSTS, which might mediate an increased attention to gestural information when speech is degraded. Subsequently, we postulate that listeners might engage their motor cortex to possibly simulate gestures more when speech is degraded to extract semantic information from the gesture to aid degraded speech comprehension, while strategic processes are implemented by the ACC to allocate attention to this semantic information from the gesture when speech is degraded. We interpret the larger alpha suppression over visual areas as a larger engagement of these visual areas to allocate visual attention to gestures when speech is degraded. In future eye‐tracking research, we will investigate how and when listeners exactly attend to gestures during degraded speech comprehension to better understand how listeners direct their visual attention to utilize visual semantic information to enhance degraded speech comprehension. We suggest that the language network, including LIFG, is engaged in unifying the gestures with the degraded speech signal, while enhanced gamma activity in the MTL suggested that the semantic information from gestures can aid to retrieve the degraded input and facilitates a matching between degraded input and top–down lexical memory traces. The more a listener's alpha and beta power were suppressed, and the more gamma power was enhanced, the more a listener demonstrated a benefit from gestures to enhance speech comprehension. Our results thus go beyond previous work by showing that low‐ and high‐frequency oscillations can predict the degree of integration of audiovisual information, also in a semantic context. Importantly, this work demonstrated a clear relationship between neural and behavioral responses during gestural enhancement of degraded speech comprehension.

## Supporting information

Additional Supporting Information may be found online in the supporting information tab for this article.

Supporting Information
