# Supplementary material for: Hearing and seeing meaning in noise: Alpha, beta, and gamma oscillations predict gestural enhancement of degraded speech comprehension
Source: Hum Brain Mapp. 2018 Jan 30;39(5):2075–87. doi: 10.1002/hbm.23987 (PMC5947738; doi:10.1002/hbm.23987)
Supplement: Supplementary file 1 — Supporting Information [file HBM-39-2075-s001.docx]

**S1. Alpha power contrast comparisons - analyses of single contrasts**

Analyses of the single contrasts of the four conditions concur with these results: In line with previous research on degraded speech comprehension (Weisz et al., 2011; Obleser and Weisz, 2012; Becker et al., 2013; Meyer et al., 2013; Strauß et al., 2014a; Strauß et al., 2014b; Wostmann et al., 2015), alpha power was increased when comparing D to C (one significant positive cluster, *p* = .02, (left)-central and temporoparietal regions, 0.7 - 2.0 s). In a gestural context, however, cluster-based permutation tests revealed that alpha power was more suppressed in response to DG than D (one significant negative cluster, *p* < .001, going from left-temporal regions in an early time window (~0.7-0.9s), visual regions in a later time window (~ 0.95 - 1.6s), and back to left-temporal regions and visual regions in the final time window (1.6 - 2.0)). Finally, alpha power was more suppressed in response to CG than C (one significant negative cluster, *p < .*001, visual regions, 0.7 – 2.0), but did not differ when comparing DG and CG (*p* = .20).

**S2. Beta power contrast comparisons - analyses of single contrasts**

In order to rule out that the observed beta suppression in this interaction effect was not due to simply seeing visible speech (i.e., lips) we compared the single conditions and found that this larger beta suppression only occurred in conditions in which a gesture was present. Beta power was more suppressed over left-temporal, motor and visual areas for DG than CG (one negative cluster, *p* < .001, 0.7 – 2.0), more suppressed over left-temporal, motor and visual areas for DG than D (one negative cluster, *p* < .001, 0.7 - 2.0 s), more suppressed over central-parietal regions in response to CG as compared to C (one negative cluster, *p* < .01, 0.7 - 2.0 s), and more enhanced over left motor areas for D than C (one positive cluster, *p* = .03, 0.95 - 1.55 s). Thus, we observed a power suppression in all contrasts containing gestural information, but not in the one contrast that only contains visible speech (in fact, here the occurence of degraded speech even caused enhanced beta power as compared to clear speech, especially over left-central sensors). This suggests that the observed suppression is driven by the gesture, but not by other visual information, such as visible speech (which was also present in all conditions).

**S3. Gamma power contrast comparisons - analyses of single contrasts**

When comparing the single conditions, cluster-based permutation tests revealed differences between DG and CG (one positive cluster, left-temporal areas, , *p* < .05, 1.0 - 1.7 s) between DG and D (one positive cluster, bilateral temporal-parietal areas and occipital regions, *p* < .05, 1.0 - 2.0 s) but not between D and C (*p =* .15) or CG and C (*p* = .21). These results suggest that gestural information might require more neuronal computation and active processing when speech is degraded and a gesture is present.
